# New Technologies and Gambling Perception Among Italian Managers: A Qualitative Study

**DOI:** 10.1007/s10899-025-10441-8

**Published:** 2025-10-23

**Authors:** Giuseppe Iraci-Sareri, Fabiano Pesticcio, Diego Fabiani, Evelina Marallo, Claudia Bianchi, Tiberio Favagrossa, Lucrezia Ballerini, Eleonora Topino, Alessio Gori

**Affiliations:** 1 Gruppo Incontro Società Cooperativa Sociale, Pistoia, 51100 Italy; 2 Integrated Psychodynamic Psychotherapy Institute (IPPI), Via Ricasoli 32, Florence, 50122 Italy; 3 Department of Health Sciences, University of Florence, Via di San Salvi 12, Pad. 26, Firenze, 50135 Italy; 4 Department of Human and Social Sciences, Mercatorum University, Piazza Mattei, 10, Rome, 00186 Italy

**Keywords:** Gambling, Smartphones, Workplace, Prevention, Managers, Risk Perception

## Abstract

The spread of new technologies has profoundly transformed gambling practices, extending their reach into both private and professional domains. Understanding how managers perceive these phenomena is essential for designing effective preventive interventions in organizational contexts. This qualitative study explored the perceptions of 33 Italian managers regarding gambling, associated risks, and the role of personal devices. Semi-structured interviews were conducted and analyzed using inductive thematic analysis. Seven themes emerged, organized into three main areas: (1) perceptions of gambling, including differences in prevalence, motivations for gambling, and perceived changes linked to digitalization; (2) perceptions of risk, highlighting both protective factors (e.g., education, awareness, controlled environments) and major risks such as social and family deterioration, economic difficulties, and illegality; (3) use of personal devices, with managers describing blurred boundaries between work and private life, alienating aspects of constant connectivity, but also perceived benefits in terms of efficiency and reassurance. The findings show that managers’ perceptions are marked by both awareness and misconceptions, which play a crucial role in shaping organizational responses. Preventive strategies should therefore be grounded in how the phenomenon is actually perceived in workplace contexts, to ensure greater acceptability and sustainability.

**Supplementary Information:**

The online version contains supplementary material available at 10.1007/s10899-025-10441-8.

## Introduction

Gambling is a rapidly growing social and public health issue worldwide (Thomas & Thomas, 2015; Deans et al., 2016). In Italy, the treasury reported gambling revenues that nearly doubled compared to 2019 (ADM, 2020, 2023).

In recent years, online gambling has become more widespread and is often engaged by very young people (ADM, 2023). A recent study showed that despite the legal impediment, 52% of Italian fifteen-year-olds have gambled in the last year, while 6.4% gamble habitually (Favieri et al., 2023).

### Gambling and New Technologies

The increase in online gambling activity has been associated with the development of new technologies that allow gaming terminals to be accessed more easily, often through mobile devices (Olason et al., 2011). It has been found that online gambling appears to be associated with a higher probability of developing pathological gambling symptoms even if the relationship is strongly mediated by the amount of time spent in gambling (Baggio et al., 2017).

Other studies that have investigated the relationship between the diffusion of new technologies (such as mobile devices) and the increase in gambling show instead that adaptation to new technologies functions as a protective factor (see Storer et al., 2009 for a review). In fact, previous qualitative research has shown a complex and nuanced association between problem gambling and the use of particular online platforms that simulate gambling without real money: while experienced gamblers appear better able to differentiate between gaming and gambling (thus, minimizing harm), these platforms may exert a problematic influence on individuals at risk (e.g. Gainsbury et al., 2015).

### The Expansion of Gambling Environments

This phenomenon extends beyond individuals’ private life. Recent qualitative research on pathological gamblers has found that the use of mobile devices enables individuals to gamble increasingly often even in the workplace (Lopez-Gonzalez et al., 2021), and this can reduce productivity (Papineau et al., 2018). This issue has become more prominent with the growing digitalization of work, which has created environments increasingly compatible with online gambling (Griffiths, 2009). From a social perspective, workplace culture may also contribute to sustaining gambling behaviours. Gamblers who receive approval or encouragement from peers are more likely to maintain their habits (Delfabbro & Thrupp, 2003). Qualitative research has further shown that the beliefs and attitudes toward gambling that circulate within a workplace can shape how gambling is experienced (Hing & Breen, 2005). Studies on employees of gaming venues, for example, have demonstrated that they are more exposed to gambling-related cues and therefore more vulnerable to developing problematic behaviours (Williams et al., 2004). At the same time, the introduction of active workplace policies aimed at preventing gambling has been suggested as a potential protective measure (Griffiths, 2009), though these initiatives are often left to the discretion of executives and managers.

### The Present Study

Previous qualitative research on the topic has already explored gambling-related embezzlement in workplaces (Binde, 2016), and other authors have reported that gambling, through the use of smartphones, has also colonized the workspace in clinical populations (López-González et al., 2020). Finally, emphasis is placed on the need to move beyond traditional “problem gambling” paradigms to explore how technological developments and processes of globalization contribute to gambling related risks (Cassidy et al., 2013). Existing studies have not addressed this issue from a managerial viewpoint, especially within the Italian context. Therefore, the present research aims to investigate managers’ perceptions of gambling, its spread in the workplace, and its relationship with new technologies such as mobile devices.

This is in order to improve knowledge on the phenomenon and be able to better guide future targeted and preventive interventions against problem gambling and behavioural addiction in organisations. This is expected to increasingly be a challenge in these new work environments, where the integration of mobile technologies and personal devices in work tasks is increasingly permeating with all the problems that derive from it (Marinas et al., 2021).

Given the exploratory nature of the study and the aim of collecting perceptions on the topic, a qualitative research design was adopted, using the thematic analysis technique. Considering the nature of the data and the absence of a precise, predefined model, this method was deemed more suitable than quantitative approaches for addressing the objectives of the present research.

## Methods

### Recruitment

The sample of this study is made up of workers who in various capacities hold a role of coordinating other employees within their respective work environments. Participation was voluntary. Workers were contacted through trade unions and trade associations, and were informed about the project’s objectives, their right to refuse participation, and the option of not authorizing the use of data. Interviews lasted on average 34 min (range: 18–52 min). All interviews were conducted in agreement with the companies during working hours, and the participants did not receive additional compensation or salary incentives for participation in the study. All the companies involved in the study are based in the municipality of Florence in Italy, but 2 of the participants report being residents of neighbouring regions and commuting.

A total number of 33 participants (10 female) was involved, of which 3 did not give consent for the interview to be recorded.

The average age is 52.9 years (SD = 9.32, min 25, max 69). Participants were asked whether they considered themselves at risk with regard to the phenomenon of gambling, using a single multiple choice question (see supplementary material), and they self-reported that they considered themselves at no risk (70%), low risk (26.7%) or high risk (3.3%) for the development of gambling disorders, furthermore only 37.5% report that among the people close to them there are people at risk.

### Procedure

The data were collected in the period between March and May 2024 using a semi-structured interview that contained both open and closed questions. All interviews were conducted in Italian (a translated version of the interview is reported to facilitate reading as a Supplementary Material), and some interviews were conducted by telephone. Prior to participation, informed consent was obtained from all participants, followed by the completion of demographic questions.

The interview was constructed based on research questions emerging from previous literature, by the project’s scientific coordinator and the scientific director who coordinated the team of interviewers (both psychologists with experience in gambling prevention). This helped focus the questions on exploring the factors that actually appear to influence the actual spread of gambling in workplace settings through new technologies. The interview was shared with the entire research team, who were able to make adjustments, primarily to improve the interview’s reception in workplace settings and to mitigate the bias in some questions, which was necessary to collect quantitative data useful for project reporting purposes.

The first part of the interview included a series of questions on the perception of gambling, what is known about it, how widespread it is, how it can be categorized. Then questions were asked about the perception of risk related to gambling in general, the risk perceived towards themselves and those close to them. At the end of this section, an open question containing dynamic norms (Reynolds-Tylus et al., 2024) was used to increase arousal on the topic and extract richer answers.

The second section focused on perceptions of device use, exploring whether it invaded private spaces, whether using devices for personal activities affected work productivity, and how new technologies have transformed both work and leisure time. Finally, a series of closed questions were presented on whether the use of these devices had created problems in the workplace or in one’s private life.

In the last section of the interview, closed questions were asked on the presence of policies within the company where they work aimed at regulating the use of devices at work or for work activities during free time, preventive policies against gambling, gambling regulations or awareness-raising activities on the topic and also whether problems have ever occurred in the workplace due to gambling or the use of personal devices. Finally, an open question was asked on the perception of a link between work stress and gambling, and an open question was left at the bottom for free considerations by the interviewee.

The main objective of the interview design was to elicit free associations between gambling and device use, while minimizing any influence on the direction of participants’ responses in order to investigate the perception of executives and managers regarding the characteristics of these phenomena and their interconnections as well as having a warning on the perception of the diffusion and severity of these issues.

### Data Analyses

Given the exploratory nature of the study and the considerable complexity and richness of the data, an inductive thematic analysis was conducted (Braun & Clarke, 2006). Interviews with participants who provided consent were audio-recorded, then transcribed. The transcriptions were read and re-read, and then coded line by line by a single researcher. The categories were composed with the ideas that appeared in multiple interviews or that were extremely relevant for the purposes of the investigation. Each interview was then re-read light of the formulated categories, which were expanded and modified accordingly. The categories were then reduced to themes following the research question.

In line with the research objectives, the questions were grouped into 3 topic groups:

1. Perception of gambling, what is known about it, how widespread it is;
2. Perception of risk related to gambling in general, the risk perceived towards themselves and those close to them;
3. Use of personal devices, both in the workplace and in private life, how these new technologies have transformed work activity and free time.

Coding was then performed separately for each group of questions, leading to the production of themes for each research question.

To ensure maximum impartiality in the analysis, coding was carried out by a research assistant with a degree in psychology and training in qualitative methods, but with no prior experience in gambling or involvement in gambling prevention contexts. This choice was intended to ensure a more impartial analysis, minimizing positional bias, despite the coding being conducted by a single researcher. Furthermore, the coder did not participate in the interviews and does not know either the interviewees or the interviewers, and did not consult with superiors regarding the composition of the themes, so as to minimize the effect of interpersonal and contextual interactions on the analysis (Jamieson et al., 2023; Olmos-Vega et al., 2023). Binary questions were used to capture polarized positions on some specific points of the analysis and were reported as descriptive where applicable.

## Results

From the thematic analysis, a total of seven themes emerged, organized according to the three research questions. Specifically:

1. Perceptions of gambling, including three themes, namely Differences in the prevalence of gambling, Opinions on why people gamble and Perceived changes in gambling;
2. Perceptions of risk in gambling, including two themes, namely Protective factors and Perceived risks of gambling;
3. Use of personal devices, including two Themes, namely Fusion between private and working life and Changes in the use of devices.

Each of which is composed of several sub-themes. The frequencies ​​reported below define the number of interviewees who spontaneously brought up the sub-theme in their interview.

### Perceptions of Gambling

This first set of questions was designed to explore participants’ knowledge and preconceptions regarding gambling and its spread (see Table 1.).

**Table 1.** Themes and subthemes of perceptions of gambling

Perceptions of gambling		Frequencies
Differences in the prevalence of gambling		**Frequencies**
Territorial differences		6
Socio-economic transversality		2
Opinions on why people gamble		**Frequencies**
Hope of earning and getting rich		5
Gambling to fill the void		2
Degeneration from socialized gambling		2
Perceived changes in gambling		**Frequencies**
The changes are due to the new devices		5
Difficulty identifying the problem		4
It is more widespread		4

I. Differences in the prevalence of gambling.

The analysis shows that there is a widespread perception regarding the transversality of the phenomenon which affects different social classes and income groups, even if it is reported that southern Italy is more exposed than the north (*n* = 4).

*<< It has a big weight especially in the poorest places […] I think like in the south where one has more time*,* there is less work and therefore one organizes differently.>>*.

Only one interviewee also believed that foreigners are more exposed than natives while another that the severity of the problem is greater in the suburbs than in the city centers. Furthermore, older people (*n* = 2) and young people (*n* = 4) are perceived to be more at risk. Only one respondent believed that women are less at risk than men.

*<< I have the feeling that this dynamic is growing among both young and old people.>>*.

II. Opinions on why people gamble.

The most frequently cited motivation was the pursuit of wealth, or the hope of winning a great amount of money and being able to change their lives, which in a more advanced stage it also adds the desperate attempt to recover losses.

*<< the seed of gambling and that psychological escape of wealth*,* of the escape from work: I’ll change jobs*,* I can’t stand it*,* so if I win I’ll go and open a beach bar>>*.

However, especially regarding the most frequent gamblers, there is the perception that they gamble to chase away bad thoughts. For example, individual interviewees reported that they think gamblers do it to relieve stress, ward off boredom, seek satisfaction, and compensate for a sense of loneliness and social marginalization. Furthermore, there is also the idea that problematic gambling may begin when socially accepted gambling activities spiral out of control.

<< *Because if someone is frustrated at work*,* and is looking for satisfaction*,* then gambling in some way…>>*.

III. Perceived changes in gambling.

Some interviewees also report perceiving changes in gambling behaviour. Gambling is perceived as a growing phenomenon, increasingly attracting young people (*n* = 4).

*<< [Gambling] has certainly passed from a marginal activity has a real problem on a social level>>*.

These changes are primarily linked by interviewees to the advent of new technologies; individual interviewees explain that these have made gambling platforms more accessible, and that through personalized marketing and the creation of niche gamblers, the phenomenon appears increasingly socialized to gamblers.

*<< Technology has accustomed*,* let’s say it facilitated these things>>*.

Furthermore, thanks to these technological developments, the phenomenon is much easier to conceal, so gamblers are perceived as capable of hiding their gambling activity even from those close to them who in this way are unable to identify the problem.

*<< If someone gambles with his personal device*,* how can you know if he is a gambler and if he is spending all his money?>>*.

### Perceptions of Risk in Gambling

In this second group of questions the interviewees were asked what their perception is of exposure to gambling with regards to the Italian context, themselves and the people close to them (see Table 2.).

**Table 2.** Themes and subthemes of perceptions of risk in gambling

Perceptions of risk in gambling		Frequencies
Protective factors		**Frequencies**
Education and training		3
Recreational gambling		2
Risk perception		2
Extremely controlled environment		2
Perceived risks of gambling		**Frequencies**
Deterioration of social and family relationships		15
Economic difficulties		8
Illegality and underground gambling		6
Loss of job		3
Disconnection from reality		2
Suicide risk		2

I. Protective factors.

In a few interviews, the perception of protective factors against gambling emerges. Among these, the most frequently cited was education and awareness about gambling. Participants believed that gaining knowledge about the risks and mechanisms of gambling could help individuals better understand and avoid problematic behaviours.

<*< Educate and also know*,* if you don’t know the problem then you can also fall into it.>>*.

Another topic was the idea that engaging in recreational or simulated forms of gambling, such as video games, board games, or social casino games, might serve as a protective alternative to gambling. Some participants described these activities as fulfilling the same recreational function without financial risk:

<<*I’ve been a serial PlayStation gamer since I was young. My free time is just that. I’ve never gotten involved [in gambling].>>*.

However, opinions on this point were mixed. A minority of interviewees expressed concerns that such activities could, in some cases, become excessive and even lead to gambling problems, particularly in vulnerable individuals (*n* = 3). At the same time, some participants argued that simply being aware of gambling risks could act as a protective factor, since perceiving oneself as at risk makes it less likely to develop pathological behaviours.

*<< I think that as long as I feel at risk I don’t fall for it*,* so it’s always better to be afraid*,* it’s a bit like smoking*,* drugs or food.>>*.

Specifically in the workplace it seems that regulated and supervised working environments also with the help of new technologies are perceived as risk protective, however in a single interview it emerged that with the evolution of gamblers’ behaviours, they are able to find ever new ways to evade control and technological innovation is slightly late to cover these strategies.

Finally, only one participant reports that legal and regulated gambling is perceived as an institutional tool of protection against illegal gambling affairs.

II. Perceived risks of gambling.

When considering risks, the main concern expressed by interviewees is the deterioration of the social and relational sphere including the compromise of family relationships, also linked to facing economic difficulties also due to the perceived increased probability of losing job, which in some cases places people in a condition of shame for their status and leads them to social isolation (*n* = 6), while in others it leads them to ask friends and relatives for money to continue betting, thus deteriorating relationships (*n* = 3).

*<< It can lead to exclusion from the context of normal life […] and from working life>>*.

A problem perceived as an extremely harmful characteristic of gambling is the presence of a dense network of illegal gambling (*n* = 5) and a loan sharking business (*n* = 2) connected to it which leads gamblers to find themselves in conditions of serious debt, this condition together with the sense of marginality and shame are perceived as causes of the risk of suicide.

*<< Gambling on something that is beyond*,* let’s say*,* the real availability of the person who gambles and therefore risks getting into debt and then perhaps even turning to individuals […] who are outside the circuits of legality.>>*.

### Use of Personal Devices (Workplace & Private Life, How Do They Change our Habits)

Participants were asked to reflect on how the use of personal devices influences both private and working life and how it has modified their daily habits (see Table 3).

**Table 3:** Themes and subthemes of the use of personal devices

Use of personal devices	Frequencies
Fusion between private and working life	**Frequencies**
Free time and work are no longer separate	9
COVID as a watershed	5
Changes in the use of devices in free time	**Frequencies**
Feeling of totalizing and alienating experiences	8
Perception of time optimization	5
Increased peace of mind by always being informed	4
Fear of being controlled	3
Perceived generational differences in device use	2

I. Fusion between private and working life.

The survey sought to investigate how private and working life interfered with each other due to the spread of new mobile devices, but from the thematic analysts it emerged that for a large percentage of people private sphere and working life are no longer perceived as separate.

*<< Once you could be on working time or you had free time*,* now it’s all much more confusing >>*.

Furthermore, it was found that this change was attributed not to mobile technologies per se, but to the measures implemented to enable agile working in response to the closures imposed during the COVID-19 emergency. The pandemic is therefore perceived as a technological watershed between a traditional concept of work and the current one (*n* = 5).

*<< Before COVID and after COVID it has really changed: relationships*,* the way of working*,* the way of approaching devices*,* everything has changed completely.>>*.

From the binary questions it emerges that 26 of interviewees believe that the use of mobile devices in the workplace influences productivity, while 28 believe that they have caused the intrusion of work into their free time.

II. Changes in the use of devices.

Participants expressed different attitudes toward these technologies, which were predominantly negative. In particular the interviewees report that constant interconnection is seen as an alienating experience that distances us from reality and engulfs people’s lives. This constant attention to online compromises not only the mood due to the constant bombardment of emotionally captivating stimuli but also relationships with other people.

*<< I have this terrible opinion*,* that social media*,* especially non-work ones*,* are becoming a parallel life or a way of showing a life that we don’t have. […] Many people no longer live their own life.>>*.

Another fear that emerged concerns the risk of constant monitoring, both in professional activity (*n* = 2), and also in private life (*n* = 2), such as through targeted marketing, or in the collection of metadata as reported by individual interviewees.

*<< there is the whole very particular issue of the abuse of devices in terms of control of operations*,* […] this should be prohibited.>>*.

But it must also be said that the half of interviewees perceive positively the possibility of using mobile devices (*n* = 15), in particular these report being relieved by the feeling of always being reachable and of being able to be contacted in real time so as not to have to worry about their loved ones during working hours.

*<< Even just having the possibility of bringing your own device and always having contact can make the person feel better*,* in my opinion it is fundamental>>*.

Others instead report that they believe that thanks to these tools they can carry out their activities in a simpler and faster way, but also that this aspect of extreme competitiveness forces the use of these devices if one does not want to remain socially and professionally marginalized (*n* = 3).

*<< …you have to be part of the system […] And the system is fast*,* to be fast you need the “smart”>>*.

From the interviews it also emerges that young people are perceived as more exposed to risk in the use of technology because they lack life experience (*n* = 2), individual interviewees believe that they are also facilitated in the use of electronic devices and that for young the telephone is not just for calling but is the key to accessing the digital world.

*<< I’m old*,* let’s say*,* and a little bit… in short*,* I try to be more in control because I’ve lived a while also without a cell phone. But are children who live like this the masters*,* when they go to the supermarket*,* to choose what they want? Or are they programmed by this society?>>*.

A considerable percentage reports that problems have occurred in their private life (*n* = 12) or in their workplace (*n* = 10) due to the use of personal devices. And that their work organizations have produced guidelines for the use of private mobile devices in the workplace (*n* = 16) and company mobile devices in their free time (*n* = 8).

## Discussion

Understanding how managers perceive both gambling and the use of new technologies is crucial for informing effective psychological and organizational interventions. These two phenomena (often treated separately) are increasingly interconnected in the modern workplace, where mobile devices enable constant access to potentially risky behaviours, including gambling. As key decision-makers, managers have a dual role: they are both observers of emerging risks and agents capable of shaping preventive policies and workplace cultures (Griffiths, 2009). However, the findings of the present research show that only 51.7% of companies have any regulations on the use of personal devices at work, and just 21.4% have implemented specific policies to address gambling. This suggests a potential mismatch between perceived risks and institutional responses, highlighting the need to better understand how such perceptions are formed and how they may influence organizational priorities.

The thematic analysis led to the identification of seven themes, distributed across the three research questions, which were considered by the managers interviewed as the most salient.

### Perception of Gambling

I. Differences in the prevalence of gambling.

Interviewees described gambling as a widespread phenomenon, often associating its prevalence with specific social, territorial, and demographic contexts. For instance, some participants perceived southern Italy as more exposed to gambling risks, an impression that partially aligns with national data showing a higher prevalence of pathological gambling in southern regions, despite relatively uniform gambling activity across the country (ADM, 2023; Pacifici et al., 2019). Participants also identified youth and older adults as particularly vulnerable populations. Although epidemiological data highlight the 25 to 64 age group as the most affected by gambling disorders (Pacifici et al., 2019), these perceptions suggest that risk is often conceptualized in terms of developmental or social fragility, rather than statistical prevalence. Interestingly, the perceived differences between urban centers and suburbs, or between genders, appeared less prominent in participants’ accounts than in national statistics. This may indicate that some aspects of gambling risk are more salient or visible to managers based on their lived experience and workplace context. These divergences highlight the importance of understanding how risk is framed and recognized in everyday organizational life, especially considering that such perceptions influence whether and how preventive actions are taken.

II. Opinions on why people gamble.

Participants highlighted several reasons why people gamble, which can be grouped into financial, emotional, and social dimensions. The most frequently mentioned motivation was the hope of earning large sums of money and radically changing one’s life, sometimes extending into the desperate attempt to recover previous losses. This motivation has been widely recognized in the literature as a key driver of gambling persistence (Tabri et al., 2022). Alongside financial motives, gambling was also described as a way to cope with psychological strain. Some respondents perceived it as a strategy to relieve stress or escape from frustration, boredom, and dissatisfaction at work. Others associated it with the need to compensate for loneliness or social marginalization. These accounts point to the use of gambling as a form of emotional regulation, an aspect widely discussed in the clinical literature. In particular, previous studies have shown how gambling can function as a maladaptive coping strategy, sometimes accompanied by dissociative states that temporarily reduce distress but contribute to the development of dysfunctional patterns (Gori & Topino, 2024; Gori et al., 2022; Topino et al., 2021, 2024). Finally, some participants emphasized that even socially accepted and recreational gambling could, over time, escalate into problematic behaviour. This perception is consistent with qualitative research showing that gambling often remains normalized until its negative consequences become evident (Reith & Dobbie, 2013).

III. Perceived changes in gambling.

Participants perceived gambling as a phenomenon that has become increasingly widespread, with particular concern for its growing diffusion among young people. These perceptions are supported by epidemiological studies documenting gambling involvement among Italian adolescents (Cena et al., 2022). Interviewees largely attributed these changes to the advent of new technologies. According to their accounts, online platforms have made gambling more accessible and visible, also through the use of personalized marketing strategies and targeted offers that foster the creation of niche groups of gamblers. These observations resonate with recent findings highlighting how digital technologies have transformed gambling environments, making them more pervasive and socially integrated (Lopez-Gonzalez et al., 2021). Another recurrent theme was the difficulty of identifying gambling problems. The possibility of gambling on personal devices was perceived as allowing individuals to conceal their activity from family members, colleagues, or supervisors, thereby reducing the chances of early recognition and intervention. This concern is in line with research showing that online gambling, compared to land-based gambling, is more easily hidden and often associated with delayed help-seeking (Gainsbury et al., 2015).

### Perceptions of Risk in Gambling

I. Protective factors.

From the interviews, several elements emerged as being perceived by managers as protective against the development of gambling-related problems. Among these, training and education were the most frequently mentioned. Some participants expressed the belief that greater awareness and understanding of gambling can help reduce individual vulnerability, a view that is also supported by previous research showing the potential benefits of educational programs (see Keen et al., 2017 for a review). Participants also discussed the idea of “recreational gambling” as a potential buffer against problematic gambling. While this was perceived in an ambivalent way (with some viewing it as a substitute for gambling, and others as a possible gateway) such ambiguity mirrors findings in the literature. Participants’ ambivalent views mirror findings in the literature showing that simulated gambling may, depending on the context and individual vulnerability, either mitigate or exacerbate risk (Gainsbury et al., 2015). Some participants suggested that awareness of the risks itself could act as a protective factor, a view consistent with research showing that individuals with greater risk perception are less likely to engage in problematic gambling (Hing & Breen, 2006). A few participants mentioned the role of environmental control, especially workplace supervision and regulation, as potentially protective. This perception aligns with studies showing that increased monitoring and structured environments can reduce the likelihood of gambling-related issues (Meyer et al., 2009; Vachon et al., 2004). Finally, one participant reported that legal and regulated gambling could act as a protective barrier against illegal gambling practices and this is consistent with the idea that regulation can reduce exposure to unregulated, higher-risk forms of gambling (Israeli & Mehrez, 2000).

II. Perceived risks of gambling.

Interviewees most frequently associated gambling with risks to the social and relational sphere. In particular, they highlighted the deterioration of family relationships, linked to financial difficulties and, in some cases, to the perceived possibility of losing one’s job. Feelings of shame and marginalization were described as consequences of these difficulties, sometimes leading to social isolation, and in other cases to requests for financial support from relatives and friends, thereby further straining interpersonal relationships. These perceptions are consistent with research emphasizing the relational impact of gambling problems, which often extend beyond the individual to affect the broader family and social environment (Dowling et al., 2016). Another recurrent concern was the link between gambling and illegality. Several participants referred to the presence of illegal gambling networks and loan-sharking activities, which were seen as exposing individuals to serious debt and exploitation. The combination of indebtedness, marginalization, and shame was perceived as contributing to extreme outcomes such as suicidal risk. This perception resonates with previous findings that underline the association between gambling-related debt, social stigma, and suicidality (Moghaddam et al., 2015; Sharman et al., 2019).

### Use of Personal Devices

I. Fusion between private and working life.

Participants described a growing overlap between private and working life, reporting that these domains are no longer perceived as clearly separate. This perception was often attributed not directly to the spread of mobile technologies, but to the reorganization of work practices during the COVID-19 pandemic, which was seen as a watershed moment that reshaped habits and expectations around digital connectivity. Similar accounts have been reported in the literature, which identifies the pandemic as a turning point in accelerating digital transformation and blurring work–life boundaries (Marinas et al., 2021). When asked about productivity, participants expressed ambivalent views. Some perceived mobile devices as tools that could facilitate task completion, while others felt that their use resulted in the intrusion of work into private time, reducing the possibility of rest and detachment. This tension reflects broader debates in the literature, which emphasize how mobile technologies can both enhance flexibility and autonomy while also contributing to role conflict and stress when boundaries are not clearly defined (Yemoh & Amitai, 2022).

II. Changes in the use of devices.

Participants expressed negative attitudes toward the pervasive use of mobile devices, which were often described as alienating and intrusive. Constant connectivity was seen as overwhelming, exposing individuals to continuous emotionally charged stimuli and compromising interpersonal relationships. These concerns echo research linking digital hyper-connectivity with reduced psychological well-being and social fatigue (Aagaard, 2015). Another recurrent theme concerned fears of surveillance, with respondents referring both to workplace monitoring and to commercial practices such as targeted marketing and data collection. Such concerns are consistent with wider debates on the psychological consequences of surveillance capitalism (Zuboff, 2019). Despite these negative aspects, many participants also reported positive experiences. Being constantly reachable was perceived by some as reassuring, particularly for staying in touch with family members, while others emphasized the efficiency and flexibility enabled by mobile devices. However, this greater efficiency was also accompanied by the perception of increased competitiveness and the risk of marginalization for those unable to keep up with technological demands. These ambivalent perceptions mirror findings that mobile technologies can simultaneously empower and burden individuals, depending on the social and organizational context (Mazmanian et al., 2013). Generational differences were also noted. Young people were seen as more adept at navigating digital environments, but at the same time more vulnerable to overuse due to their limited life experience. This dual perception reflects findings that younger generations are both empowered and exposed by their immersion in digital contexts (Livingstone & Helsper, 2007). Finally, participants reported that conflicts related to mobile device use occurred both in private life and in the workplace. In response, several organizations had introduced guidelines regulating the use of personal devices during work hours and company devices during personal time. These initiatives reflect a growing awareness of the need to set boundaries for digital engagement (Farivar et al., 2022; Mondo et al., 2023).

#### Limitations and Suggestions for Future Research

The present study provides interesting ideas for intervention, yet several limitations should be acknowledged. Given the very low percentages for some themes, it is possible that saturation was not reached with this number of interviews, and therefore the topics with lower percentages were not fully explored in this study. Furthermore, the sample was limited to the municipality of Florence (Italy), which restricts the generalizability of the findings. Voluntary participation may have introduced self-selection bias, as those most interested in the topic were more likely to take part. Additionally, the gender composition of the sample was not balanced, resulting in a predominantly male perspective on the phenomenon. In some questions it was necessary to use a leading language to delve into specific topics. In addition, some dichotomous questions were used to obtain partially quantitative data, which required participants to adopt polarized positions before elaborating their views. These factors may have influenced the quality of the collected answers, exposing to risks of bias and conditioning. Moreover, the exploratory design with a qualitative method implies that the results may be influenced by the researchers’ subjective interpretations. Finally, the use of a single self-perception item to assess participants’ gambling risk represents a further limitation. Although this approach was chosen to minimize interview duration and respondent burden, it does not provide clinically validated information.

Future research should aim to address these limitations through several methodological improvements. First, incorporating standardized and validated instruments would enable a more accurate and objective assessment of gambling risk. Second, adopting quantitative or mixed-method research designs could offer a more robust understanding of the relationship between new technologies and gambling behaviours in the workplace. Third, expanding the sample across broader geographic regions and occupational roles would enhance the external validity of findings. Given the relevance of age in shaping attitudes toward both gambling and digital technology, future studies should also explore generational differences, comparing how younger and older workers perceive and respond to these evolving risks.

## Conclusions

This study explored how managers perceive the intersection between gambling and new technologies in both professional and personal contexts, with the aim of identifying actionable insights to support preventive policies in organizational settings. The themes that emerged from the interviews reflect a complex mix of awareness, concern, and misconception. These perceptions offer a meaningful lens into how gambling and digital device use are socially interpreted within the workplace. Some views expressed by participants suggest a limited recognition of the risks associated with gambling in working-age populations. The tendency to frame gambling as a problem primarily affecting youth or older adults may lead to the underestimation of risks within employee groups who are, in fact, highly exposed. Addressing this perception gap could be a strategic focus for interventions, helping managers to reframe the issue as relevant to their own organizational reality (Hertwig, 2017). Similarly, while some participants emphasized the dangers of constant connectivity and digital distraction, others viewed mobile technology as a source of reassurance, autonomy, or even social necessity. These divergent perceptions indicate the importance of tailoring interventions that acknowledge both risks and perceived benefits, promoting a balanced digital culture rather than enforcing strict control measures (Mazmanian et al., 2013). Overall, the findings suggest that effective interventions should not only disseminate information, but also engage with managers’ existing representations, including their emotional and moral interpretations. By starting from how the phenomenon is actually perceived, rather than how it should be understood according to external models, policies and training initiatives can be made more acceptable, targeted, and sustainable within organizational life.

## Supplementary Information

Below is the link to the electronic supplementary material.

Supplementary Material 1(DOCX 33.0 KB)

## Declarations

### Data statement

The data presented in this study are available on request from the corresponding authorThe data presented in this study are available on request from the corresponding author.
