# Supplementary material for: New Technologies and Gambling Perception Among Italian Managers: A Qualitative Study
Source: J Gambl Stud. 2025 Oct 23;42(2):697–714. doi: 10.1007/s10899-025-10441-8 (PMC13269313; doi:10.1007/s10899-025-10441-8)
Supplement: Supplementary file 1 — Supplementary Material 1(DOCX 33.0 KB) [file 10899_2025_10441_MOESM1_ESM.docx]

| **Part 1. This section is about your perception of the phenomenon of gambling.** | | | | | |
| --- | --- | --- | --- | --- | --- |
| 1) | What do you know about the phenomenon of gambling? | | | | |
|  | | | | | |
| 2) | How widespread do you think gambling is? | | | | |
|  | | | | | |
| 4) | | What is gambling for you?  a) A vice b) A pastime c) An illness d) A fun e) A way to make ends meet f) A recreational activity g) Other _________________________ | | | |
| 5) | | | On a scale of 1 to 10, where 1 = “Not at all” and 10 = “A lot,” how much do you think the use of devices for gambling activities can lead to distraction from work? | | |
|  | | | \| 1 \| 2 \| 3 \| 4 \| 5 \| 6 \| 7 \| 8 \| 9 \| 10 \| \| --- \| --- \| --- \| --- \| --- \| --- \| --- \| --- \| --- \| --- \| | | |
| 6) | | | What do you think are the main risks of gambling? | | |
|  | | | | | |
| 7) | | | Are there any people close to you who you consider to be at risk for gambling? | **yes** | **no** |
| 8) | | | In your opinion, how much does this phenomenon weigh on today's society? | | |
|  | | | | | |
| 9) | | | In Italy, 36.4% of adults gamble, while in Tuscany it is 38%, what do you think of these estimates? | | |
|  | | | | | |
| 10) Regarding the phenomenon of gambling, do you consider yourself a person with:   1. No Risk b) Low Risk c) Moderate Risk d) High Risk | | | | | |
| If b), c) or d) is chosen, investigate further with respect to the perception of risk. | | | | | |

| **Part 2. This section concerns your perception of the use of electronic devices (personal and otherwise) that are used during working hours and during extra-work activities.** | | | |
| --- | --- | --- | --- |
| 11) | In general, do you believe that the use of devices (smartphones, tablets, computers) for personal use in the workplace can influence workers' productivity? | **yes** | **no** |
|  | If yes, please specify |  |  |
| 12) | In general, do you think that the use of devices (smartphones, tablets, computers) during free time can influence the quality of one's life outside of work? | **yes** | **no** |
|  | If yes, please specify |  |  |
| 13) | On a scale of 1 to 10, where 1 = “Not at all” and 10 = “A lot,” how much do you think the use of devices for extra-work activities can lead to distraction from work? | | |
|  | \| 1 \| 2 \| 3 \| 4 \| 5 \| 6 \| 7 \| 8 \| 9 \| 10 \| \| --- \| --- \| --- \| --- \| --- \| --- \| --- \| --- \| --- \| --- \| | | |
| 14) | On a scale of 1 to 10, where 1 = “Not at all” and 10 = “A lot”, to what extent do you think the use of devices for extra-work activities can lead to an intrusion of work into your life outside of work? | | |
|  | \| 1 \| 2 \| 3 \| 4 \| 5 \| 6 \| 7 \| 8 \| 9 \| 10 \| \| --- \| --- \| --- \| --- \| --- \| --- \| --- \| --- \| --- \| --- \| | | |
| 15) | In your opinion, how much is the average time spent online during working hours, over the course of a working day, via devices for extra-work activities?  a) Less than 30 minutes b) Between 30 minutes and one hour  c) Between one hour and one and a half hours d) Between two and three hours  e) Between three and four hours f) More than four hours | | |
| 16) | In your opinion, what is the average amount of time spent online during free time, over the course of a working day, using devices for work-related activities?  a) Less than 30 minutes b) between 30 minutes and an hour  c) between an hour and an hour and a half d) between two and three hours  e) between three and four hours f) more than four hours | | |
| 17) | What do you think about the diffusion and how devices are used in the workplace? | | |
|  |  | | |
| 18) | What do you think about the diffusion and how devices are used in free time? | | |
|  |  | | |
| 19) | Have you noticed any changes between before and after the diffusion of these technologies in the organizational realities in which you have worked? | **yes** | **no** |
| If yes, please specify | | | |
| 20) | Have you noticed any changes between before and after the diffusion of these technologies in your free time? | **yes** | **no** |
| If yes, please specify | | | |
| 21) | In your opinion, how widespread is the use of personal devices during working hours in your organization? | | |
|  | | | |
| 22) | In your opinion, how widespread is the use of personal devices during free time in your organization? | | |
|  | | | |
| 23) | Have there been any problems (e.g. discussions, absenteeism, etc.) in your organization related to the use of devices in the workplace? | **yes** | **no** |
| 24) | In your private life, have there been any problems (e.g. arguments, avoidance of social activities, etc.) related to the use of devices for work activities during non-work hours? | **yes** | **no** |
| 25) | In your opinion, how are devices used most in the workplace for extra-work activities?  a) Browsing online in general b) Using Social Networks c) Gambling online  d) Watch movies e) Search for information f) Communicate with friends  g) Organizing your free time | | |
| 26) | In your opinion, how are devices used most in free time for work activities?  a) Communicating with colleagues b) search the internet/keep informed c) Check the email d) Communicating with customers e) Organize the work activity of the following days  f) Other_______________________________________________________________ | | |

| **Part 3. This section concerns the organization and its prevention/information activities to combat the phenomenon of gambling and the dysfunctional use of devices.** | | | |
| --- | --- | --- | --- |
| 27) | Does your organization have any guidelines or regulations regarding the use of personal devices during work hours? | **yes** | **no** |
| If so, how were they made? | | | |
| 28) | Does your organization have any guidelines or regulations regarding the use of work-related devices outside of working hours? | **yes** | **no** |
| If so, how were they made? | | | |
| 29) | Are there or have there been any critical situations related to gambling in your organization? | **yes** | **no** |
| If so, what measures were taken to address the phenomenon? | | | |
| 30) | Are preventive policies in place in your organization related to gambling and the use of devices for personal purposes? | **yes** | **no** |
| 31) | Do you think there could be a link between work stress and gambling addiction? | **yes** | **no** |
| 32) | If you were to compare your organization with other similar companies (e.g. companies operating in the same sector or adopting similar organizational styles), how would your company rank: | | |
| - Regarding the risks associated with gambling? | | | |
|  | | | |
| - With respect to information and prevention policies on gambling? | | | |
|  | | | |
| 33) | Are there any considerations you would like to add that could enrich our investigation? | | |
|  | | | |

We thank you for your time and cooperation.
